# A Qualitative Exploration on Perceived Socio-Cultural Factors Contributing to Undernutrition Among Under-Fives in the Southern Highlands of Tanzania

**DOI:** 10.3389/ijph.2023.1605294

**Published:** 2023-07-21

**Authors:** Gasto Frumence, Yannan Jin, Amalberga A. Kasangala, Mary A. Mang’enya, Saidah Bakar, Bertha Ochieng

**Affiliations:** ^1^ Department of Development Studies, School of Public Health and Social Sciences, Muhimbili University of Health and Allied Sciences, Dar es Salaam, Tanzania; ^2^ Leicester School of Allied Health Sciences, De Montfort University, Leicester, United Kingdom; ^3^ Department of Preventive Services, Health Promotion Section, Ministry of Health, Dar es Salaam, Tanzania; ^4^ Department of Community Health, School of Public Health and Social Sciences, Muhimbili University of Health and Allied Sciences, Dar es Salaam, Tanzania; ^5^ Centre for Primary Care Research, Faculty of Health and Life Sciences, De Montfort University, Leicester, United Kingdom

**Keywords:** under nutrition, under-fives, cultural practices, social factors, Tanzania

## Abstract

**Objective:** Under nutrition especially among under-fives is a major public health challenge in Tanzania. However, the contribution of cultural practices to child under nutrition is often overlooked. This study aimed to explore the perceived socio-cultural factors contributing to the persisting under nutrition among under-fives in Tanzania.

**Methods:** The study applied focus group discussion (FDGs) with forty practitioners to examine the sociocultural factors contributing to under nutrition during early childhood. The study participants were purposively selected and thematic analysis was used to identify themes within the data.

**Results:** This study revealed that, under nutrition for under-fives is caused by a number of socio-cultural factors including existence of gender inequality related to dietary practices and qualities, women’s excessive workload, patriarchy social-norm, excessive alcohol use and cultural taboos prohibiting women and girls from eating certain types of nutrient dense foods.

**Conclusion:** The study highlights the multiplicity of factors including socio-cultural perspectives contributing to under nutrition among under-fives, and calls for a concerted efforts in developing and implementing issue-specific and culturally sensitive strategies towards elimination of child under nutrition.

## Introduction

Globally, childhood undernutrition is one of the leading causes of under-five morbidity and mortality. It is indicated by wasting, stunting, underweight, and deficiencies of micronutrients, commonly iron, zinc, iodine, and vitamin A. Undernutrition is mainly caused by an inadequate dietary intake of energy and nutrients, the presence of disease, or the interaction of both [1]. It contributes to 45% (3.1 million) of preventable deaths among children under five, as mostly found in low- and middle-income countries (LMIC) [2]. In 2020, it was estimated that globally 149 million children under five were stunted, with a high prevalence in Africa where 45 million were wasted [3]. It was reported that 219 million (39%) children under five in LMICs were at risk of not reaching their developmental potential [4].

In Tanzania, for example, a high prevalence of undernutrition for children under five in the form of stunting (>40%) and underweight (>15%) persists in several regions [5]. Notably, the Southern and Southwestern highland regions of Tanzania have a greater rate of stunting and underweight above the national average of 34.4% [6], despite the fact that they are the major food producers for the nation. Quantitative studies have suggested that factors such as children being born in rural areas, a low education level of the mother, poor maternal nutritional status, impoverished households, and large family size may contribute to an increased risk of child undernutrition, particularly stunting [7, 8].

For decades, several organizations such as UNICEF and the WHO have implemented nutrition-sensitive and nutrition-specific interventions in the afflicted LMICs that target both the immediate and underlying determinants of child malnutrition such as food and nutrient intake, feeding and caregiving, and poverty and food insecurity in order to improve child growth and development. However, the outcomes from both types of intervention are mixed and the evidence generated is not robust but limited [9]. Culturally informed practices can interplay with other factors including socioeconomic status and the level of education. Yet, the contribution of sociocultural influence to the determinant factors of child nutrition is often overlooked in nutrition interventions and recommendations. A recent example is the Global Syndemic of Obesity, Undernutrition, and Climate Change. Their concept of a planetary healthy diet advocates for less meat consumption [10], yet cultural aspects are not considered in their recommendations. Due to the complexity surrounding the implementation of culturally sensitive interventions to the target population, most of the studies to date have paid less attention to cultural issues.

Cultural aspects are highly variable, yet with a degree of overlap across different socio-class structures, ideologies, and ethics [11]. Culture can influence and transform how an individual or a group of people behaves toward power dynamics and social relations in their lives. Cultural contexts can also shape the food accessibility and dietary quality of the mother pre- and post-partum and further influence decision-making on breastfeeding and complementary feeding practices [12]. Moreover, culture shapes how men engage in maternal and child nutrition, and healthcare and spouses are sometimes left out in matters pertaining to Infant and Young Child Feeding (IYCF) practices [13].

Cultural practices related to maternal and child nutrition vary greatly across regions and ethnic groups in Tanzania. They start early before conception, yet with the most impact exerted throughout pregnancy. Examples of these practices include forbidding pregnant women from eating certain foods, particularly those high in protein and fat, food myths and fallacies like sugar cane consumption being believed to be linked to child drooling, involuntary starvation, and self-induced morning vomiting, among many others that compromise maternal nutrition and health, negatively affecting pregnancy outcomes like stillbirths and low-birth-weight babies.

Moreover, some of these cultural practices for pregnancy are extended to the period of lactation together with other types specifically introduced after giving birth. Examples include delayed or lack of initiation of breastfeeding within the first hour of life, pre-lacteal feeds, discarding colostrum, early initiation of complementary feeding, and the use of traditional medicine for illnesses [14, 15], leading to poor child nutrition, growth, and development, the consequences of which are seen later in life.

The available evidence has shown that a low education level of the mother, poor maternal nutritional status, impoverished households, and large family size all account for an increased risk of childhood undernutrition in the Southern Highland regions of Tanzania. However, little has been documented regarding how cultural practices from different parts of the Southern Highlands contribute to the current situation, in which all five regions have above the national average prevalence rate of child stunting (34.4%), that is, Njombe (53.6%), Rukwa (47.9%), Iringa (47.1%), Songwe (43.3%), and Ruvuma (41.0%). Despite the fact that these five regions constitute the most important production area of food crops in Tanzania, they have the highest prevalence of child stunting [6]. The unresolved issues invite a timely exploration of the socio-cultural role in causing undernutrition among children under five in the Southern Highland regions in Tanzania.

## Methods

### Study Design

The study employed an exploratory study design in gathering evidence regarding the perceived social cultural factors surrounding maternal and child nutrition from key people coordinating nutrition activities in the study areas. Four Focus Group Discussions (FGDs) were used to collect data from regional- and district-level nutrition health coordinators, and experts and stakeholders on nutrition issues from different organizations including the Ministry of Health (MoH), the President’s Office-Regional Administration and Local Government (PO-RALG), academic and research institutions, and government agencies in Tanzania. The workshop aimed at discussing existing socio-cultural practices and how they have contributed to undernutrition for under-fives in the Southern Highlands regions of Tanzania and suggest possible culturally sensitive strategies to combat the child undernutrition issue.

### Sample Size

About 40 participants including regional- and district-level nutrition health coordinators, officials from the MoH and PO-RALG, and researchers and academicians attended the 1-day expert workshop to discuss the cultural practices and other factors that may cause chronic undernutrition in children, the associated long-term health and socioeconomic effects, and collectively propose culturally sensitive strategies to improve the nutritional status in early childhood. During the workshop, participants were divided into four Focus Group Discussion (FGDs), each consisting of 10 participants.

### Sampling Strategy

Participants were purposively sampled from the regional- and district-level nutrition health coordinators, national level officials including those from the MoH, PO-RALG, and government agencies, and researchers, academicians, and NGOs interested in nutrition issues. All the participants were selected based on the roles they play in implementing and coordinating various nutrition-related interventions which influence the efforts towards the reduction of undernutrition among under-fives at the family, community, and district levels.

### Ethical Consideration

The study was approved by the Ethical Review Board of the Muhimbili University of Health and Allied Sciences (MUHAS). There was no identified form of harm to the respondents. Participants signed a consent form before data collection and were reassured that their information would be kept confidential.

### Data Collection

Data collection started with a brief workshop in which the participants were firstly briefed about the aim and objectives of the study. Thereafter, they were invited to participate in the FGDs; all gave informed consent, then four focus group discussions were conducted with each group containing 10 participants. An FGD guide was developed in English and translated into Kiswahili to facilitate data collection, and was pretested before using it for actual data collection. The guide consisted of open-ended questions regarding the underlying cultural practices leading to the persisting child undernutrition issues. Each focus group lasted between 60 and 90 min and all were audio-recorded. During the conduct of the FGDs, one researcher, assisted by a research assistant (RA), administered the FGD, including taking summaries of key issues that emerged during discussions. The saturation point of the emerging themes were reached in the fourth FGD whereby there were no new themes that were emerging by continuing to hold more FGDs.

### Data Analysis

All interview transcripts were transcribed verbatim and translated from Kiswahili into English, followed by thematic data analysis and inductive reasoning. Emerging themes across a sample of transcripts were identified and validated by three researchers before carrying out a line-by-line analysis. The use of an inductive approach was sought to ensure that the emerging themes were strongly linked to the data, rather than being imposed by the researcher [16, 17]. The audio-recorded interviews were transcribed and translated from Kiswahili into English. Two coders coded the data and the analysis was done manually in three steps: line-by-line coding of transcripts, in which emerging concepts were developed; the examination and interpretation of codes into descriptive themes; and condensation of descriptive themes into more abstract analytical themes.

## Results

### Socio-Demographics

A total of 40 key stakeholders in nutrition issues participated in the FGDs (Table 1). Of the 40 study participants, 20 (50%) were aged between 35 and 44 years, and 24 (60%) were female. In total, 32 (80%) study participants had a college/university level of education. Among the FGDs participants, seventeen (42.5%) were nutrition officers, four (10%) were medical doctors, five (12.5%) were nurses, and six (15%) were health officers.

**TABLE 1:** Socio-demographic characteristics of study participants (Tanzania, 2021).

Characteristic	National level (*N* = 20) Number (%)	Regional level (*N* = 20) Number (%)	Total (*N* = 40) Number (%)
Sex			
Male	9 (45)	7 (35)	16 (40)
Female	11 (55)	13 (65)	24 (60)
Age (years)			
25–34	4 (20)	4 (20)	8 (20)
35–44	9 (45)	11 (55)	20 (50)
45–55	4 (20)	4 (20)	8 (20)
>55	3 (15)	1 (5)	4 (10)
Level of education			
Certificate	3 (15)	3 (15)	6 (15)
Diploma	1 (5)	1 (5)	2 (5)
Bachelor’s degree and above	16 (80)	16 (80)	32 (80)
Cadre			
Medical doctor	2 (10)	2 (10)	4 (10)
Health officer	2 (10)	4 (20)	6 (15)
Nurse	1 (5)	4 (20)	5 (12.5)
Nutritionist	11 (55)	6 (30)	17 (42.5)
Others (administrator, pharmacist)	4 (20)	4 (20)	8 (20)

### Qualitative Themes

The analysis of qualitative data generated several themes illustrating the socio-cultural causes of child undernutrition. The themes were: the unequal distribution of power between men and women, unequal food distribution during family meal times, existence of food taboos, lack of decision-making power for certain health and nutrition services among women/girls, and the existence of alcohol addiction for both men and women. The following section briefly presents the analysis of these themes representing the main socio-cultural causes of child undernutrition in the study area.

### Unequal Distribution of Power Between Men and Women

Socio-cultural factors are the larger scale forces within cultures and societies that affect the thoughts, feelings, and behavior. Such factors include: attitudes, childcaring practices (which include feeding practices), family structures, regional differences, and taboos. Among the socio-cultural factors that were discussed and given much weight in all FGDs was patriarchy, which refers to the unequal distribution of power between men and women in certain aspects of the societies. In these male-dominated societies, all decisions with regards to family income such as the selling of crops is handled by the husband/spouse. The use and distribution of household resources such as food is decided by the man of the family. The man may decide that providing his family with quality diets or sending them for health services are not his priorities and thus his family will eventually suffer from lack of a balanced diet or delay in accessing health services, which in turn may lead to child undernutrition.

*“In Rukwa, we produce many foodstuffs. Every cereal is produced in Rukwa. Also, we produce fruits and keep chicken but you find high levels of child undernutrition. When I say male-dominance that means during farming, the whole family is involved. During selling only the father is involved. It is not surprising to find no stock of foodstuffs in the house but the father is drinking beer and* *eating barbecue in groceries* *. This leads to starvation and stunting of children.” (Male participant, Rukwa)*

Another FGD participant said:

*“Men own land, and women ask for permission regarding which produce is to be grown on such land; he also decides the sales of what women have produced, such as livestock and agricultural products as well as deciding how to spend the income.”* *(Female participant, Iringa)*

### Unequal Food Distribution During Family Meal Times

Gender inequality was also associated with certain dietary habits that may have a negative impact on the nutritional status of women and children. Participants from Rukwa reported a tradition that is practiced in their societies where women and girls eat what is left by men and boys. This tradition prevents women and children (in this case girls) from getting adequate food and necessary nutrients, which is likely to contribute to undernutrition for girls in some of the families.

*“The way people eat in towns is very different from villages. You will find the lion’s share is for the father and usually there is very little food for women and children. The lion’s share is for the husband and male children and the food for women is inadequate. I* *think this discipline has been passed down, that females should respect males, which leads to undernutrition. Let me also say it is not only that they eat last but also the distribution of food as pointed out. The* *father is given lots of food, almost three times more than that taken by women and girls.” (Female participant, Rukwa).*

### Existence of Food Taboos

Participants from different regions spoke about food taboos and how this affects the nutrition of women and children. For instance, women and girls are not allowed to eat smelly foods such as fish and eggs, which are great contributors of essential nutrients to a child’s healthy development.

One of the participants noted that:

*“If children miss important nutrients from eggs and fish, what do you expect? They will definitely suffer from undernutrition and their growth will be highly compromised.” (Male participant, Songwe).*

It was also reported that women and girls are not allowed to pick vegetables from a garden during their menstrual periods. This has been a traditional practice affecting household vegetable intake because the family members have to wait until the woman/girl completes her menses.

The following quote substantiates the finding above

“*There is misconception that ladies are not allowed to pick vegetables in the garden while on her period. If there is nobody else to pick the vegetables, then the household would not consume vegetables on that day.” (Female participant, Rukwa*)

### Lack of Decision-Making Power for Certain Health and Nutrition Services Among Women/Girls

Moreover, another cause of child undernutrition was reported to be the poor utilization of family planning services. If a woman uses family planning methods, she will be considered a harlot; with this fear, women end up having many children and, with the struggle of caring for them, the roles are delegated to older siblings, leading to inadequate and poor childcare practices, which may cause undernutrition to children since children are not given a balanced diet.

Study participants also reported that there are false beliefs that undernutrition is caused by witchcraft. Some parents attribute the undernutrition of their children to the acts of witchcraft, thus taking their children to traditional healers like herbalists for treatment, which in turn aggravates the problem of undernutrition because of delays in taking children for medication at health facilities. One of the participants narrated as follows:

*“Undernutrition in some parts of the district is still high because of misconceptions that witchcraft causes undernutrition and men decide to take their children to traditional healers.” (Male participant, Rukwa).*

The general understanding gained in the study was a lack of nutrition knowledge among fathers which contributes to the causes of undernutrition in children under five. Fathers were reported as having economic power in the family, thus making them responsible for ensuring their female partners and children all get nutritional foods. However, the findings show that they have little or inadequate knowledge of types of nutritional foods to purchase or prepare for their newborns. Since children are not given nutritional food, they end up suffering from undernutrition.

*“Most the fathers do not have knowledge of nutrients required to feed their children. Some have little knowledge. This situation largely contributes to undernutrition since fathers possess economic power at the household level.” (Male participant, Iringa).*

### Existence of Alcohol Addiction for Both Men and Women

Alcohol addiction was reported among fathers and mothers that reduced the quality of caring given to their children. Furthermore, women complained that men take a long time at beer stores and they return home so late in the day that they have no adequate time to care for their pregnant partners or their newborns. On the other hand, it was reported that in some regions many women are alcohol addicts and they pass childcaring practices to their children while they spend hours drinking local brew. The study participants noted that if children are not well cared for, they will not be given nutritional food, thus, they will suffer from undernutrition.

*“Another thing is alcoholism for both men and women, which makes them sometimes lose sense of control and fail to sit together and discuss nutrition issues.” (Male participant, Iringa)*

## Discussion

This study identified several socio-cultural factors contributing to child undernutrition in Tanzania. Findings demonstrated the existence of gender inequality related to eating practices and nutritional qualities that may have a negative impact on women and children’s—especially girls’—nutritional status. For instance, a tradition still practiced in some of the regions involves unequal food distribution during meal time, and also women and girls eating only after men and boys. In most cases, such traditions prevent women and girls from getting adequate food and necessary nutrients, which contributes to the persistent child undernutrition in some of the study areas. A similar tradition is cited in Saudi Arabia, where men and boys eat before women and girls. Also, in these patriarchal societies, males are rarely involved in the caring duties for children, only the mothers are [18]. Women’s workload was also flagged by the participants as a cause for child undernutrition as women are involved in multiple activities like farming, fetching firewood and water, and performing all household chores solely without support on a daily basis, leaving them little or no time for proper childcaring practices. Despite the fact that all the regions in the study areas are large producers of food in Tanzania, most of the children in these regions end up getting inadequate nutrients because their parents or caregivers do not practice proper childcaring, which eventually contributes to child undernutrition. Similar findings have been reported in Rwanda [19], Bangladesh [20], and Uganda [21], where the local cultures expect women to multi-task and handle the load, deeming related tasks as feminine. Male involvement in supporting women so they can get adequate time for feeding and childcare is advocated in this context.

Other food taboos that restrict women and girls from eating certain foods were also mentioned as a cause for undernutrition. For instance, women and girls are restricted from eating foods that are considered “smelly,” such fish, eggs, and other foods like milk and liver which are rich in key macro- and micronutrients such as calcium and protein essential for children’s growth [22, 23]. Others reported that women and girls are not allowed to pick vegetables from gardens during their menstrual periods. This prevents them from consuming vegetables rich in micronutrients such as folate and non-heme iron, leading to an inadequate nutrient intake and impaired health status in a long run. In South Africa, similar findings reported that cultural taboos or beliefs made women and girls avoid eating certain types of foods such as eggs, fish, meat products, potatoes, butternut, fruits, beans, and pumpkin, which are rich in essential micronutrients, protein, and carbohydrates. Most of these foods were avoided because the society associated them with poor pregnancy and labor outcomes, and the possibility of having an undesirable body form for the baby [24].

The unique finding of this study is the practice of masculinity and how it contributes to child undernutrition. In this case, men dominate the decision-making process in food resource allocation and health-service use at the household level. Women are the ones spending most of their time on farming activities; however, after harvesting the crops, it is men who make decisions regarding selling most of the produced food crops, while sometimes leaving very little food for the family. Men may also spend money generated from selling the food crops on alcohol. All these male-dominated practices contribute to shortages of food and disbursable money for the family, resulting in the undernutrition of women and children. In an attempt to find solutions to this problem, a study conducted by Ochieng [25] on the determinants of dietary diversity and the potential role of men in improving household nutrition in Tanzania reported similar practices and recommended that men contribute toward improving household nutrition security by reducing the consumption of food away from the home.

In this study, men were reported to have low or no knowledge of the energy and nutrient requirements of pregnant women and children. This situation constrained men from purchasing nutritional foods for pregnant mothers and children. A study in Northern Ethiopia found that fathers’ nutrition knowledge and education was an important attribute to the level of dietary diversity achieved among children [26]. This implies that nutrition knowledge among men is key to ensuring optimal nutrition outcomes for children in a household, particularly given the fact that men possess the economic and decision-making power at the household level in most African societies.

Despite the fact that there have been several achievements over the years regarding the reduction of stunting in Tanzania [6], our study findings have revealed that in some of the regions, undernutrition is still high because of the false beliefs regarding witchcraft, making it difficult for health and nutrition interventions to be deployed in some of the districts. Similarly, a study on the maternal perceptions of factors contributing to severe undernutrition among children in a rural African setting reported that there are still local cultural beliefs about witchcraft in Africa that are contributing to severe undernutrition [27].

Alcohol consumption for both men and women was reported to contribute towards aggravating the child undernutrition issues at the household level. Mothers who are addicted to alcohol do not have time to care for their pregnancy and babies while men who frequently consume alcohol deprioritize their financial spending (e.g., on food purchases) and time for their families. Similar findings were reported in South Africa, where the use of tobacco and alcohol among mothers were linked with adverse health consequences during pregnancy and for the offspring, such as poor growth and development [28]. In South Delhi, India, fathers’ alcohol abuse was also reported as a factor causing undernutrition for under-fives [29].

### Study Limitations and Strengths

The selection of nutritionists and other key stakeholders in nutrition health as study participants without including community members may contribute to missing the inclusion of ideas from community members who are engaged in day-to-day socio-cultural practices. However, the inclusion of nutritionists and other key stakeholders who have stayed and interacted with communities for a long period have given them enough experience to provide valuable insights into the socio-cultural factors contributing to undernutrition in the under-fives in the community. The possibility of social desirability bias in FGDs with regional-, district-, and national-level nutritionists may also limit this study, as they formed 42% of the study participants. However, the involvement of other study participants, including those from implementing government and non-governmental agencies, academicians, and researchers, contributed to the strengths of this qualitative study.

### Conclusion

This study revealed that in the Southern Highlands of Tanzania there are several socio-cultural factors contributing to the undernutrition for under-fives. The unique finding of this study is the existence of gender inequality related to dietary practices, qualities, and masculinity, where men make decisions without involving their partners, especially in selling crops without leaving adequate food stock for the family. Furthermore, women’s excessive workload—characterized by multiple domestic and economic roles—excessive alcohol use, and cultural taboos or beliefs prohibiting women and girls from eating certain types of nutrient dense foods such as eggs and fish were also mentioned as socio-cultural factors contributing to undernutrition among under-fives in the Southern Highlands of Tanzania. Key stakeholders in nutrition nationwide need to make concerted efforts in forming and implementing issue-specific and culturally sensitive strategies towards the elimination of child undernutrition for under-fives.

## Data Availability

Derived data supporting the findings of this study are available from the corresponding author GF on request.
